# Supplementary material for: Causal Relationship between Aging and Anorexia Nervosa: A White-Matter-Microstructure-Mediated Mendelian Randomization Analysis
Source: Biomedicines. 2024 Aug 16;12(8):1874. doi: 10.3390/biomedicines12081874 (PMC11351342; doi:10.3390/biomedicines12081874)
Supplement: Supplementary file 1 [file biomedicines-12-01874-s001.zip › Supplementary Figure/Figure S7/Single SNP plot of MR analysis for ubm-b-1749 to AN.pdf]

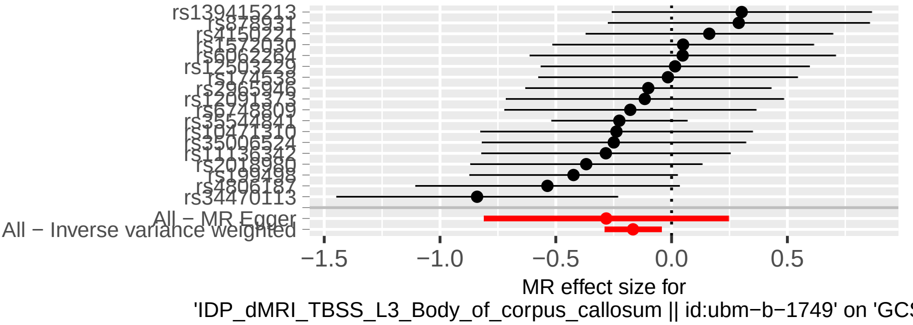
rs139415213
rs878931
rs4150221
rs1572030
rs6062264
rs12503229
rs174538
rs2965946
rs12091373
rs6748809
rs35544841
rs10471310
rs35006524
rs11136342
rs2018980
rs199498
rs4806187
rs34470113
All − MR Egger
All − Inverse variance weighted
−1.5
−1.0
−0.5
0.0
0.5
MR effect size for
'IDP_dMRI_TBSS_L3_Body_of_corpus_callosum || id:ubm−b−1749' on 'GCS
